# A large deletion on CFA28 omitting *ACSL5* gene is associated with intestinal lipid malabsorption in the Australian Kelpie dog breed

**DOI:** 10.1038/s41598-020-75243-x

**Published:** 2020-10-26

**Authors:** Mitchell J. O’Brien, Niek J. Beijerink, Mandy Sansom, Sarah W. Thornton, Tracy Chew, Claire M. Wade

**Affiliations:** 1 School of Life and Environmental Sciences, Faculty of Science, University of Sydney, Camperdown, NSW 2006 Australia; 2 Sydney School of Veterinary Science, Faculty of Science, University of Sydney, Camperdown, NSW 2006 Australia; 3 Veterinaire Specialisten Vught, Reutsedijk 8a, 5264 PC Vught, The Netherlands; 4 Sydney Informatic Hub, University of Sydney, Camperdown, NSW 2006 Australia; 5 Present Address: Callicoma Kelpies, Grafton, NSW 2460 Australia; 6 Present Address: Unaffiliated, Los Altos, USA

**Keywords:** Animal breeding, Development, Inbreeding, Mutation, Genotype, Genetic predisposition to disease, Clinical genetics, Genetic testing, Population genetics, Genetic variation, Rare variants, Structural variation, Genetics, Genetic association study, Genome-wide association studies

## Abstract

Inborn errors of metabolism are genetic conditions that can disrupt intermediary metabolic pathways and cause defective absorption and metabolism of dietary nutrients. In an Australian Kelpie breeding population, 17 puppies presented with intestinal lipid malabsorption. Juvenile dogs exhibited stunted postnatal growth, steatorrhea, abdominal distension and a wiry coat. Using genome-wide association analysis, an associated locus on CFA28 (P_raw_ = 2.87E^−06^) was discovered and validated in a closely related population (P_raw_ = 1.75E^−45^). A 103.3 kb deletion NC_006610.3CFA28:g.23380074_23483377del, containing genes Acyl-CoA Synthetase Long Chain Family Member 5 (*ACSL5*) and Zinc Finger DHHC-Type Containing 6 (*ZDHHC6*), was characterised using whole transcriptomic data. Whole transcriptomic sequencing revealed no expression of *ACSL5* and disrupted splicing of *ZDHHC6* in jejunal tissue of affected Kelpies. The *ACSL5* gene plays a key role in long chain fatty acid absorption, a phenotype similar to that of our affected Kelpies has been observed in a knockout mouse model. A PCR-based diagnostic test was developed and confirmed fully penetrant autosomal recessive mode of inheritance. We conclude the structural variant causing a deletion of the *ACSL5* gene is the most likely cause for intestinal lipid malabsorption in the Australian Kelpie.

## Introduction

Long chain fatty acids (LCFA) are the most abundant fats in mammals and play a key role in the canine (*Canis lupus familiaris*) diet. Pancreatic lipases are largely responsible for the hydrolysis of triglycerides into glycerol and fatty acids, which are absorbed across the brush border of jejunal enterocytes. Activation of fatty acids is the first step in intracellular metabolism of LCFA. The process involves the conjugation of fatty acids with coenzyme-A (CoA) and is catalysed by a group of enzymes called Acyl-CoA synthetases (ACS)^1–3^. Thirteen homologous ACS genes that activate LCFA have been annotated in mammals and cluster in three different gene families: acyl-CoA synthetase long chain (ACSL), acyl-CoA synthetase bubblegum (ACSBG) and fatty acid transport proteins (FATP)^1–3^*.* The full extent of each gene on normal intestinal absorption of LCFA is unknown. Animal models with heritable phenotypes of intestinal lipid malabsorption provide an opportunity to elucidate this.

The Kelpie is an iconic dog (*Canis lupus familiaris*) breed established in the late nineteenth century for its natural working ability and resilience in the extreme weather conditions of Australia^4^. Since inception, the breed has become divided into two separate breeding populations maintained by different pedigree registries^5^. Dogs selected primarily for strong working ability are known as the Australian Working Kelpie (AWK). The Australian Kelpie (AK) is selected according to a conformation breed standard and is registered by the Australian National Kennel Council. The Federation Cytological International recognizes both breed varieties enabling the populations to co-mingle but, in Australia, the two are maintained as separate breeding populations and are genetically distinct; most notably in genes influencing morphology and behaviour^4,5^.

We describe an inherited intestinal lipid malabsorption (OMIA 002226-9615) in the AK. Unlike cases of similar disorder hereditary selective ileal cobalamin malabsorption^6–11^, AK show no signs of lethargy and have clear evidence of fat in faeces (steatorrhea). Based on the observed familial segregation the observed phenotype is a fully penetrant autosomal recessive inherited metabolic disorder. The aim of the current study is to provide insight into the molecular genetic aetiology of intestinal lipid malabsorption in the AK.

## Materials and methods

### Ethics

The research described conforms to the recommendations from the Australian Code for the Care and Use of Animals for Scientific Purposes. Animal ethics approval was granted by the Animal Ethics Committee at the University of Sydney (approval numbers 2015/902 and 2018/1449).

### Animal selection and phenotype selection

**Figure 1:**
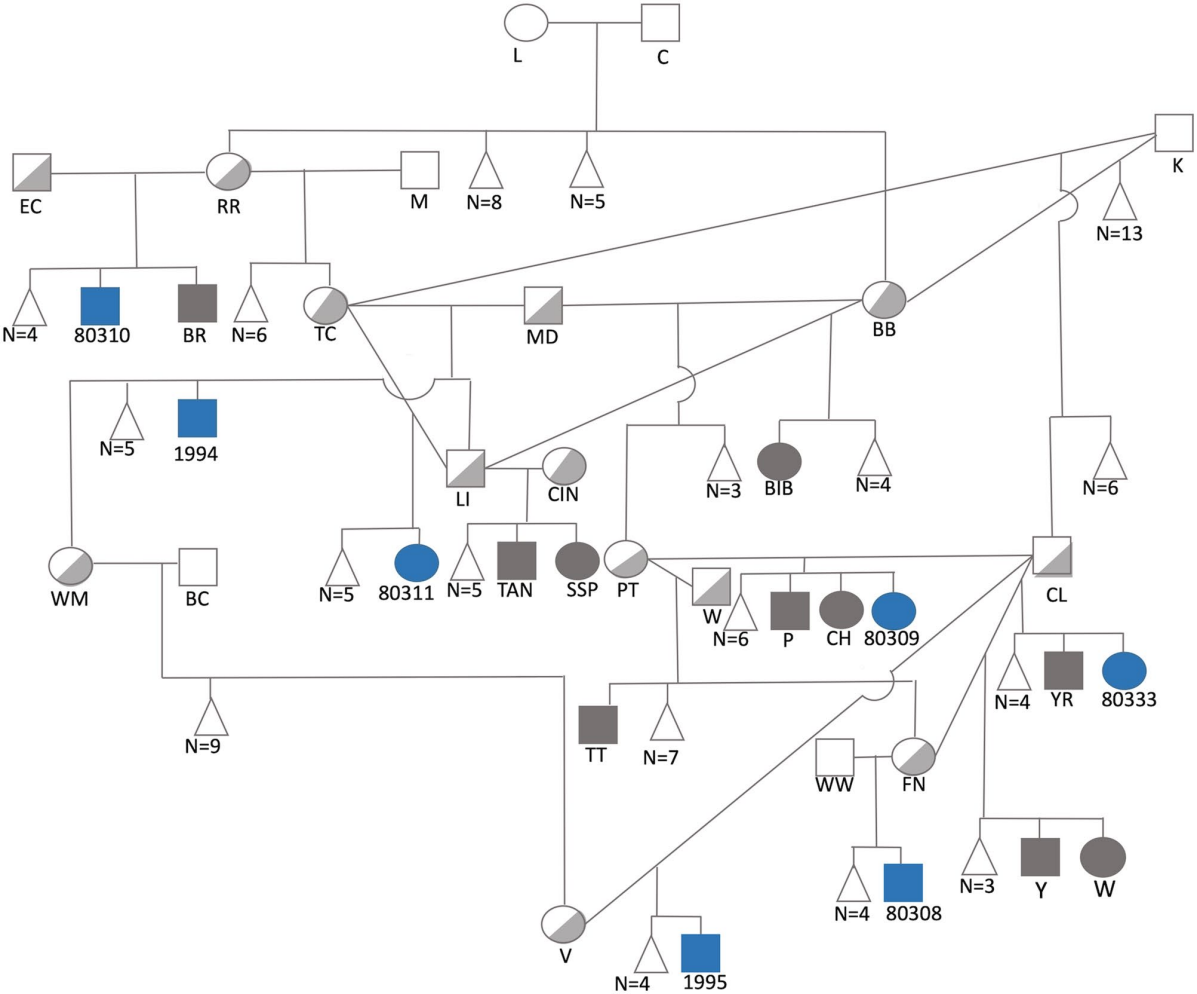
Simplified pedigree of an AK family showing links among affected individuals with hereditary intestinal lipid malabsorption. Females and males are indicated by circles and squares, respectively. Filled symbols indicate affected samples, half-filled symbols represent carriers of the disease allele based on autosomal recessive inheritance. Offspring from a single litter are represented by a line descending from a horizontal connection between parent symbols. A triangle has been used to designate multiple samples (N) from a single litter that are not affected or suspected to be carriers based on recessive inheritance. Litters that included zero affected samples have not been included. Affected samples highlighted in blue were included in the study. Diagnostic testing found all samples to be homozygous for the disease-associated variant.

**Figure 2:**
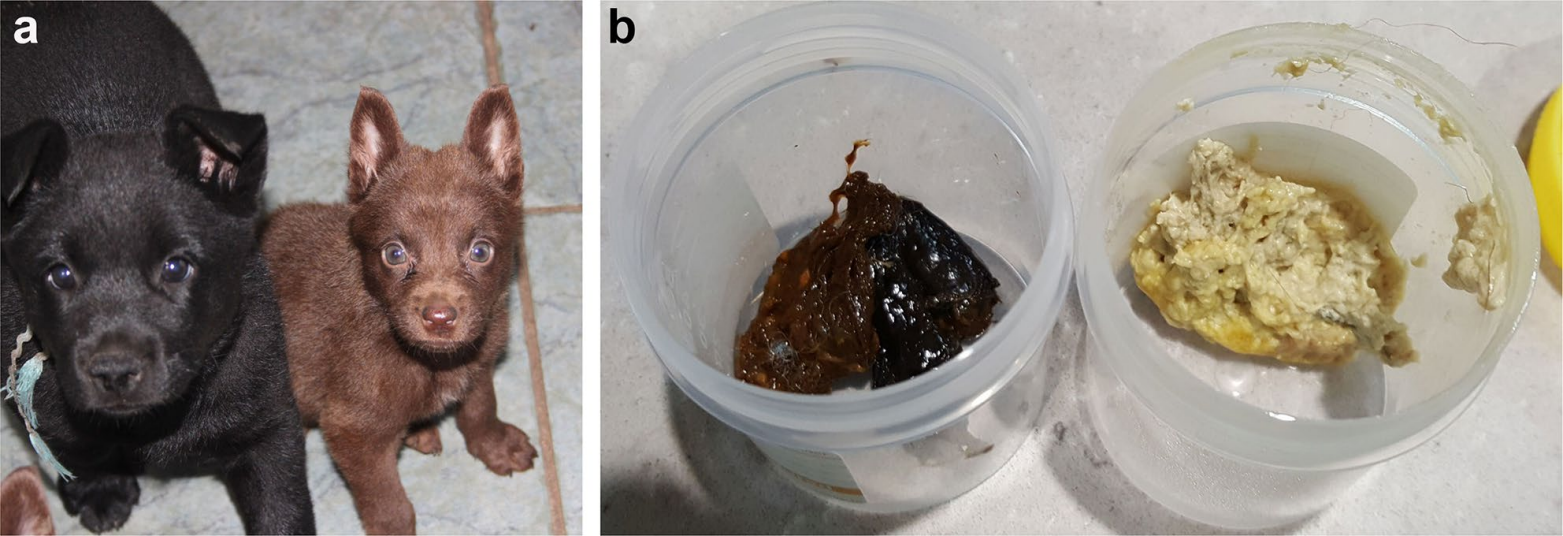
Side by side comparisons of affected Kelpie and unaffected littermate size and faecal matter. (**a**) Affected Kelpie at 10 weeks (right) with his littermate. Size and musculature of the affected pup is in stark contrast. (**b**) Pale poorly digested faeces from an affected Kelpie (right) in comparison with an unaffected littermate (left). There is a significant difference in colour and consistency between the two samples.

Related juvenile AK dogs were observed to exhibit stunted postnatal growth and intestinal lipid malabsorption. Affected individuals remain a third to one half the size of their littermates during development and mature so that adult dogs are smaller in stature and exhibit persistent intolerance to a fatty diet. Starting in 2011, 17 of 319 puppies, from 45 litters, were born at one Australian kennel presenting with identical clinical features (Fig. 1). As neonates, affected puppies are indistinguishable from their littermates, but rapidly show clinical signs of polyphagia, failure to thrive, stunted growth (around one-third to one-half of the size of their siblings—Fig. 2a), yellowish poorly digested loose and pulpy faeces (Fig. 2b), increased faecal volume, and frequent defecation. Once affected puppies are transferred to a solid diet with digestive enzyme supplementation, faecal consistency normalises. From around six months of age, most affected Kelpies appear to outgrow the characteristic clinical presentation. However, the dogs remain smaller in stature than their siblings, consistently produce more voluminous faeces than age-matched dogs, and their intolerance to high-fat foods persists throughout their lives.

This study involved 265 Kelpies. Samples were made up of 35 AK (10 cases and 25 controls), 225 AWK (225 controls), and 5 international Kelpies (one case and four controls). Cases in this study represent dogs that adhered to the described clinical presentation. Cases were easily recognised through signs of ill thrift, faecal appearance (steatorrhea), and stunted growth when compared to littermates. Seven cases from the originally described kennel have been included in this study. Two samples from separate kennels were reported as dams of affected pups. They were included as control samples and treated as obligate carriers when observing results.

Biological samples were collected as whole blood in EDTA tubes or buccal cells using cheek swabs. Genomic DNA was extracted using the PureLink Genomic DNA Mini Kit (Invitrogen, Carlsbad, CA, USA) or submitted as EDTA blood to the genotyping service provider on Whatman Flinders Technology Associates (FTA) cards, supplied by the genotyping service. Genotyping array data for 255 samples was obtained from the CanineHD BeadChip (Illumina, San Diego, CA, USA) by Neogen (Lincoln, NE, USA).

A full post-mortem was conducted at the Veterinary Pathology Diagnostic Services (University of Sydney, Camperdown, NSW, Australia) on a 17-week-old affected AK pup that was euthanized with approval by the owner on welfare grounds. A thorough examination was conducted on tissue of the lung, spleen, liver, heart, major cardiac vessels, lymph nodes, thyroid gland, kidney, bone marrow, pancreas, small intestine (duodenum, jejunum and ileum), brain and spinal cord.

### Genome-wide association study (GWAS)

To detect and validate signals associated with malabsorption in the Kelpie population two case–control GWAS were performed using Plink 1.9 (--assoc)^12^. Quality control of genotypic data was conducted on 25 AK, five internationally bred Kelpies, and 225 AWK. Single Nucleotide variants (SNVs) were excluded if they exhibited a call rate of less than 90% (--geno) or a low minor allele frequency < 10% (--maf). Pairwise identity by decent was calculated (--genome) to detect and remove duplicated or highly related individuals. Population stratification was visualised using a multidimensional scaling (MDS) plot with two dimensions (--mds). One sample from each pair with a pairwise identity by decent > 0.7 was excluded. This was done to control for inflation resulting from cryptic relatedness and population stratification. Population stratification in the preliminary GWAS was determined by the genomic inflation factor based on the median Chi-squared statistic. The primary GWAS was conducted using 30 Kelpies, including 25 AK and five internationally bred Kelpies. Both groups show evidence of carrying the studied trait; reflected in our dataset. To control for the testing of multiple hypotheses, genome-wide significant and suggestive thresholds were Bonferroni-corrected, 5 × 10^–7^ (Bonferroni cut-off of *α* = 0.05, n = 99,326) and 1 × 10^–5^ (Bonferroni cut-off of *α* = 1.0, n = 99,326), respectively. Reported *P*-values are chi-square allelic test *P*-values as calculated in Plink. The 200 most associated markers from the unstratified preliminary GWAS were taken forward to a second analysis that added 225 control dogs from the closely related population of AWK.

### Confirmation of deletion by polymerase chain reaction (PCR)

A large segment of consecutive uncalled array markers was observed only in cases suggesting the presence of a large deletion in these animals. Primers designed using primer3^13–15^ were used to detect the presence of the deletion. The novel deletion was confirmed through amplification of the last coding exons in impacted genes by PCR. Where no amplification was observed, to gauge the size of the deletion, further primers were designed to amplify the preceding exon. Alternatively, where amplification was witnessed, we designed primers in the gene’s untranslated region (UTR). A total of seven primers were designed (Table S1). PCR was carried out in total volume of 20 μl using AmpliTaq Gold 360 Master Mix (Applied Biosystems, Foster City, CA, USA) according to the manufacturer’s protocol. Fragments were evaluated on a 1.5% agarose gel. Briefly, the PCR conditions were heat activation for 10 min (mins) at 95 degrees Celsius (°C); and 30 cycles of 30 s (s) at 95 °C, 58 °C and 72 °C for denaturation, annealing and extension respectively. The process concluded with a final elongation step at 72 °C for 10 min.

### RNA sequencing, alignment and variant detection

In order to gauge if mapped candidate genes were influencing the observed phenotype, whole transcriptomic sequencing was conducted on whole tissue of the jejunum collected during post-mortem. Using Invitrogen TRIzol Reagent, RNA was extracted according to the manufacturer’s protocol. Total RNA sequencing (RNAseq) was performed on Illumina NovaSeq S1 using the TrueSeq Stranded RNA RiboZero Gold (h/m/r) kit at the Ramaciotti Centre for Genomics (University of New South Wales, Kensington, NSW, Australia). A total of 142,658,896, 100 base pair (bp) paired end reads were generated. Tissue matched transcriptomic sequence data for the Labrador retriever JEJUNUM_LABR (Accession identifier: SRR3727723) were obtained from the Sequence Read Archive (SRA) in Genbank (https://www.ncbi.nlm.nih.gov/sra/).

Quality control for the raw paired-end reads was performed with FastQC v0.11.8 (https://www.bioinformatics.babraham.ac.uk/projects/fastqc/) and visualised using MultiQC^16^. Raw RNAseq reads were mapped to the canine reference genome (CanFam3.1) using STAR aligner v2.7.0e basic options^17^. Reads surrounding the candidate region were visualised and extracted for a genome-guided de novo transcriptome assembly using Trinity v2.8.3^18^. The distribution of read data was calculated across known gene features using RSeQC v3.0.1^19^.

STAR aligned bam files and Trinity constructed fasta sequences were visualised in Integrative Genomics Viewer v2.8.2^20^. Variants in the affected Kelpie transcript were compared with those of the tissue matched Labrador retriever. Alternate transcript splicing was visualised using a sashimi plot created using ggsashimi^21^. The minimum read coverage at splice junctions was set to 15 reads to reduce background hybridisation signals.

### Multiplex PCR assay design for deletion

Custom primers were designed to capture a disease-associated variant identified in the sequenced AK using primer3. A three-primer multiplex PCR was designed to detect the structural variant (Table S1). The multiplex PCR includes a forward primer, 37 bp upstream of the disease-associated variant, and two reverse primers, one 140 bp downstream from the start of the variant and another ~ 103.7 kb downstream of the forward primer. PCR was carried out in total volume of 20 μl as previously described. A total of 19 Kelpie samples from affected populations were assessed using this method, including nine cases and ten controls. This encompassed two cases and eight controls also utilised in the GWAS analysis. Six of the controls were known to come from families that have produced affected puppies.

### Equipment and settings

All images have been formatted for publishing using Adobe Photoshop 2020 (v21.1.1). Images that have been cropped have been done so to improve clarity and conciseness. As such, all images correctly represent the original data. If an electrophoretic gel images has been cropped, it is stated in the figure legend and an original image has been provided in “Supplementary materials”.

## Results

### Post-mortem results

Post-mortem was conducted on a young, 17-week old, female Kelpie. The dog was received in excellent post-mortem condition immediately following euthanasia. The affected Kelpie pup showed signs of malnutrition including decreased body condition (body condition score 2/5), atrophied musculature and depleted subcutaneous adipose tissue stores. Histological examination of the small intestine showed evidence of mild non-specific chronic enteritis including focal ileal ulceration, rare crypt abscesses in the ileum and colon, and possible crypt fusion in the jejunum.

### Genome-wide association study (GWAS)

After frequency and genotype pruning, 99,326 SNVs remained in the analysis. Three cases and 27 control Kelpies were available for the primary analysis. Of these, four controls and one case were bred outside Australia. By MDS the AK and International populations clustered closely and so were treated analytically as one population (Fig. S1a). When AWK were included in the MDS the principal Kelpie population and AWK clustered separately (Fig. S1b).

**Figure 3:**
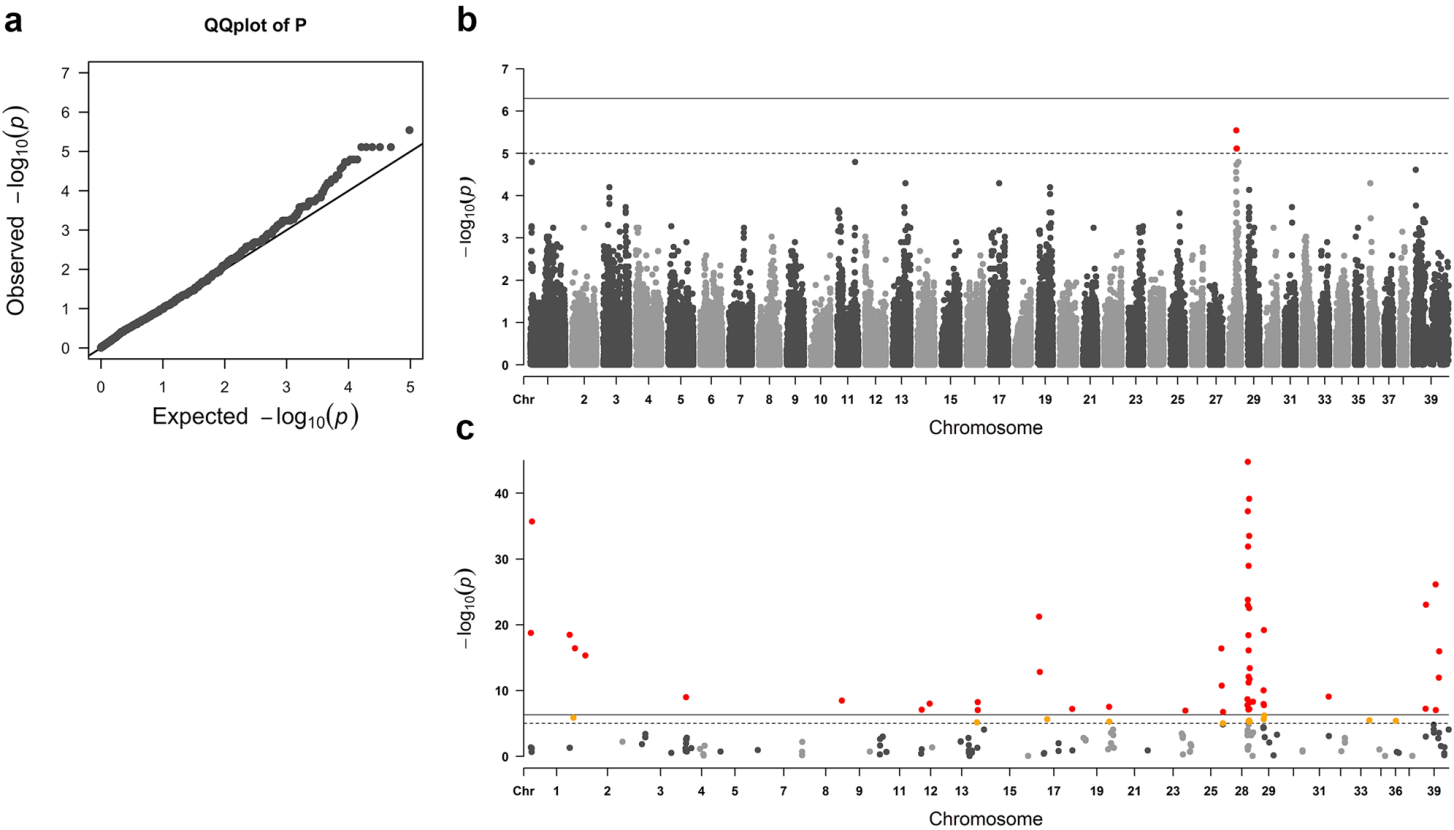
QQ-plot and Manhattan plots from intestinal lipid malabsorption case–control GWAS. (**a**) QQ-plot of Data shown in the Manhattan plot. The observed distribution of the test statistic closely follows the expected. (**b**) Manhattan plot showing the negative log P-value between individual markers. 6 SNVs highlighted in red on CFA28 passed a suggestive threshold (1 × 10^–5^, dashed line). No SNVs achieved a Genome wide significant score (5 × 10^–7^, solid line). The top 200 SNVs from this association were were taken forward to a second analysis. (**c**) Manhattan plot demonstrating genotype association to intestinal lipid malabsorption. 52 SNVs highlighted in red pass the significance threshold of P < 5 × 10^–7^ (solid line), while 13 SNVs in yellow passed a suggestive threshold of P < 1 × 10^–5^ (dashed line). The most significant region of interest is noted on CFA28, where the strongest SNV from the preliminary analysis persisted in the validation set (28:24521377 best P_raw_ = 1.75E^−45^).

A preliminary GWAS was performed in the closely clustered Kelpie populations with affected samples (AK and internationally bred Kelpies). The quantile–quantile plot shows limited inflation and the genomic inflation factor was 1.23 (Fig. 3a). GWAS revealed a suggestive association with intestinal lipid malabsorption on canine chromosome 28 (CFA28) (best P_raw_ = 2.87E^−6^) (Fig. 3b). Six SNVs within a three megabase (Mb) region (28:24,521,377–26,556,336; 2.03 Mb) passed the suggestive genome-wide significance threshold and were in strong linkage with the index SNV (r^2^ > 0.93). In the validation analysis, 225 AWK controls were added to the leading dataset. When analysing the top 200 associated SNVs in the primary GWAS in the extended cohort, 52 SNVs passed genome-wide significance (Fig. 3c). Of these SNVs, 21 (40.3%) were located on CFA28: 20 that clustered within a four Mb region (28: 24,030,090–27,194,500; 3.16 Mb) including 16 (30.7%) that matched the expected GT frequency for a recessively inherited trait (Table S2). The top SNV from the preliminary analysis remained the strongest in the validation set (best P_raw_ = 1.75E^−45^).

### Confirmation of deletion by PCR

Within the associated locus, we identified a region of nine consecutive SNVs spanning marker CFA28:23,370,822 (BICF2P674000) to CFA28:23,493,334 (BICF2P338375) 122.5 kb, where all but two SNVs were consistently uncalled in cases but not controls (Table S3), suggesting the presence of a deleted segment. Within the putatively deleted segment, three genes and one pseudogene were identified, being Tectorin beta (*TECTB*), Acyl-CoA Synthetase Long Chain Family Member 5 (*ACSL5*), Zinc Finger DHHC Domain-Containing Protein 6 (*ZDHHC6*) and pseudogene Guanylate Cyclase 2G (*GUCY2GP*). The gene *ACSL5* represented a strong regional candidate for disease. The orientation of the genes within the region of uncalled markers were positioned so that the last coding exon of each gene aligned with the edges of the putative deletion (Fig. S2a). Using seven primer pairs, we confirmed the presence of a deletion in the affected Kelpies between 101.6 kb and 105.2 kb (Fig. S2b). The PCR confirmed a complete loss of *GUCY2GP* and *ACSL5* and partial loss of *ZDHHC6*. RNAseq data was used to validate this result.

### Variant detection and RNA expression

**Figure 4:**
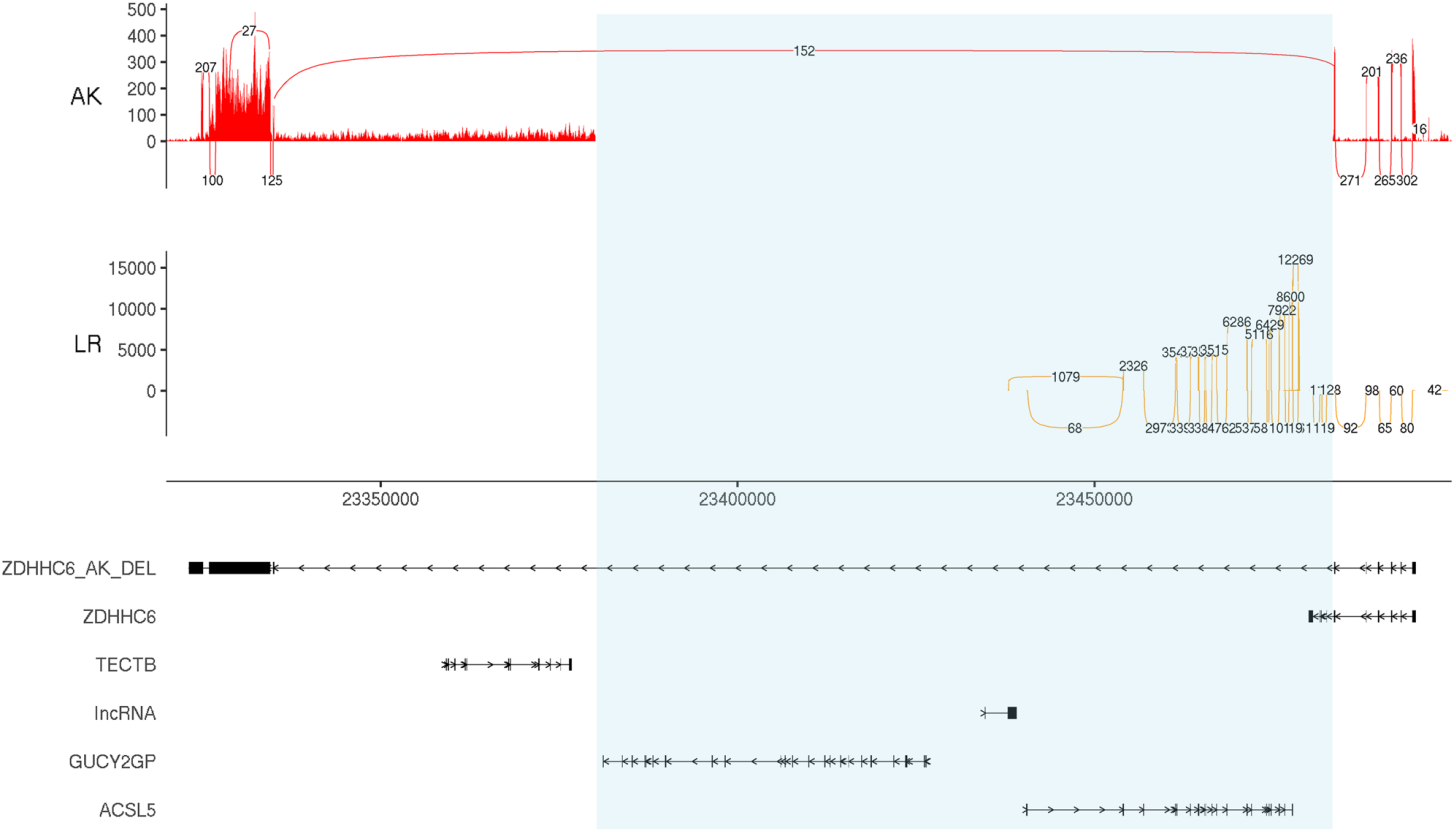
Sashimi plot of RNAseq data for CanFam3 genomic coordinates CFA28:23320000-23500000. The coverage for each alignment track is plotted as a bar graph, the Y axis represents read counts. Arcs are supported exon junctions and reads split across the junction (junction depth). Below the plots are the gene annotations for corresponding genomic coordinates. The figure illustrates RNAseq data from the jejunum of two samples; A case sample (*AK* Australian Kelpie) and control (*LR* Labrador retriever). Underlying DNA contamination can be seen in the AK highlighted by the low read count across the genomic region. A 103.3 kb deletion is seen in the AK, illustrated with a transparent box. The gap in the AK includes *GUCY2GP*, *ACSL5*, *ZDHHC6* and a Long non-coding RNA (lncRNA). In the AK, expression of novel exons can be seen 148.4 kb downstream from the *ZDHHC6* gene, the junction is supported by 152 reads. A consensus sequence produced using a genome guided de-novo assembly with Trinity is included in the gene track as ZDHHC6_AK_DEL.

RNAseq data were inspected in Integrative Genomics Viewer. Read distributions indicated RNAseq data had underlying DNA contamination with an equal portion of reads aligning to introns or intergenic regions compared to exons, 34.9% and 38.1% respectively. DNA from the AK with intestinal lipid malabsorption harboured a 103.3 kb deletion, NC_006610.3CFA28:g.23380074_23483377del (CanFam 3.1; Fig. S3), involving the complete loss of *ACSL5,* pseudogene *GUCY2GP* and omitting exons 7–10 of *ZDHHC6* (Fig. 4). RNAseq data demonstrated no detectable expression of *GUCY2GP*, *ACSL5* and *ZDHHC6* exons beyond the breakpoint of the observed deletion. A further gene, *TECTB*, located outside the deleted region had no observable expression compared with that of the Labrador retriever jejunum. Gene expression of *ZDHHC6* and *ACSL5* in the control jejunum was consistent with the reference transcript. *GUCY2GP* and *TECTB* were not expressed in either case or control. In the AK, expression of novel exons as a result of cryptic splicing were observed 148.4 kb downstream from the *ZDHHC6* gene. The alternate splicing event was captured by 152 reads. A consensus sequence was produced using a genome guided de-novo assembly with Trinity (Data S1).

### Multiplex PCR assay for deletion

A multiplex PCR test was customised to implicate the associated variant in affected individuals as well as detect carriers of the variant through allele specific amplification. Primer 1 and 3 were designed to amplify the region spanning the disease-associated variant. The variant removes 103,303 bp between the primers and results in a 414 bp fragment in carrier and affected individuals. Primer 2 anneals shortly downstream from the start of the deleted region so that wild type dogs produce a 177 bp fragment while dogs homozygous for the variant show no amplification. Animals that are heterozygous for the disease-associated variant produce both fragments. Of 19 samples tested, nine were homozygous for the deletion, all of which exhibited signs of disease (Fig. S4). In the controls, six samples were homozygous wild type and four were heterozygous for the variant. Dogs heterozygous for the variant were asymptomatic but came from families known to produce offspring with the disease phenotype.

## Discussion

Inborn errors of metabolism (IEM) are genetic disorders resulting from defects in biochemical pathways that can have a profound effect on an animal’s overall health^22,23^. IEM affecting intermediary metabolic pathways are often recognised through clinical signs such as failure to thrive, hypotonia and functional decompensation^22,23^. Increased prevalence of IEM among specific breeds has previously been observed^22–24^. Frequently reported metabolic disorders clinically similar to intestinal lipid malabsorption are hereditary selective ileal cobalamin malabsorption and exocrine pancreatic insufficiency. Both are IEM that present with failure to thrive and persistent diarrhea^11,25,26^, however the AK presents earlier (before six weeks of age), show no signs of lethargy and have clear evidence of fat in faeces (steatorrhea). Here we present an IEM affecting lipid absorption in the AK resulting from the deletion of *ACSL5* and partial loss of *ZDHHC6*.

Characterisation of the genetic factors associated with IEM is of strong interest for improving canine welfare and improving our understanding of the genomic control of metabolism. Genes influencing the phenotype described in this study, *ACSL5 an*d ZD*HHC6,* have not been previously implicated in naturally occurring disease models. Long chain acyl-CoA synthetases are major enzymes in fatty acid metabolism^27–32^. In human and rodent studies, variation in the *ACSL* gene family are often associated with diet induced metabolic and body composition phenotypes^27,31,33–38^. *ACSL5*, essential for lipid metabolism and fat deposition in carnivores^39^, is a principal candidate for the observed phenotype in the AK. ACSL genes have already been implicated in canine body composition phenotypes, with variation in *ACSL4* associated with heavy weight dogs^40^.

The clinical phenotype associated with absent expression of *ACSL5* in the jejunal tissue of affected AK puppy is consistent with a knockout (KO) mouse model, including delayed fat absorption and a reduced fat mass^27^. KO mice exhibited additional increased lean mass and energy expenditure, as well as improved insulin sensitivity; traits not observed or tested in our cases. The results of the mouse KO study contradicted an earlier *ACSL5* knockout study, which showed little effect on long-term dietary LCFA absorption and weight gain, likely compensated by residual ACSL activity^41^. Long chain fatty acid absorption occurs largely through the jejunum where LCFA are absorbed across the brush border of jejunal enterocytes. *ACSL5* is expressed in brown adipose tissue, small intestine, liver^27,28,42–44^ and is the primary activator of dietary LCFA in the jejunum^41^. Expression, synthesis and activity of *ACSL5* is connected to the state of villus architecture, epithelial homeostasis and enterocyte apoptosis^45–47^. The relatively improved health status of affected AK at maturity may imply an important role of *ACSL5* during early development. The extreme effects identified in immature AK may be partially offset by other ACSL genes as they reach full size or may be linked with a transition to a solid diet.

Following absorption of LCFA in enterocytes, they undergo re-esterification before transportation and storage is possible. Previous research in rodent studies has implicated *ACSL5* in fat absorption during the re-esterification of dietary fats^3,27,28,48^. In the present study it has been noted that once affected Kelpies are on a solid diet with enzyme supplementation, dogs continue to present with a low body condition score. While AK display ongoing sensitivity to dietary lipids into adulthood it remains unconfirmed if their smaller size is a result of persistent intestinal lipid malabsorption or stunted early development.

Further to the complete loss of *ACSL5*, the genomic deletion resulted in the partial deletion and cryptic splicing event downstream of the last translated exon of *ZDHHC6*. *ZDHHC6* plays a role in posttranslational modification (palmitoylation) of proteins, which can contribute to protein function and regulation beyond underlying genomic architecture. Differences in the palmitoylation of proteins involved in fat and carbohydrate transport and signalling may compromise digestion. Articles reviewing the biological effects of protein palmitoylation have anticipated a functional role in lipid and glucose metabolism^49,50^, though *ZDHHC6* is not currently implicated. *ZDHHC6* localises in the endoplasmic reticulum and is reported to be involved in the palmitoylation of five protein targets^51–56^. Within the context of existing research neither *ZDHHC6* nor proteins palmitoylated by *ZDHHC6* are expected to play a major role in lipid digestion. However, novel roles and targets of palmitoylation are frequently reported and the list of proteins that undergo palmitoylation is constantly growing^57^ (https://swisspalm.epfl.ch/). It is possible that other key substrates influencing the observed phenotype in the AK are not yet reported and AK harbouring the disease-associated variant may be a unique tool in furthering our current understanding of post-translational modification.

*TECTB* and *GUCY2GP* were not expressed in either the case or control RNAseq samples. The genomic region containing the *TECTB* transcript falls outside the observed variant. It is unlikely that gene expression is altered in appropriate tissue samples. Mice studies indicated that *GUCY2G* plays a role in jejunal integrity^58^. However, *GUCY2G* is a known pseudogene in humans and was suggested to be under purifying selection in the dog^59^. Conversely, Ensembl genebuild predicts the transcript is non-protein coding (Gene identifier: ENSCAFG00000010908), and recent canine gene catalogue observing ten tissue types reported no expression across all samples and replicates^60^. The gastrointestinal tract was not reported in the catalogue but a lack of expression in the Labrador retriever control supports the concept of *GUCY2GP* as a pseudogene, indicating no involvement in the observed phenotype.

Therapies to overcome deficit in *ACSL5* function are currently unknown and were not assessed in this research. In humans, therapies for disorders disrupting lipid digestion and absorption, involve removing lipids from the diet or replacing them with those that bypass the genetic block^61,62^. The disorder described in this study chiefly impacts the metabolism of LCFA. Some human studies have demonstrated positive effects of medium-chain triglyceride formulation (MCT) on individuals suffering from long chain fatty acid disorders^63–65^, however the use of MCT in canine research is restricted^66–69^. Auxiliary research into therapeutic options especially during early development is necessary.

Results of the multiplex-PCR were consistent with a fully-penetrant autosomal recessive disorder. Results reported here are not indicative of breed-wide prevalence rates as dogs included in this study originated from a small group of Australian kennels. However, the presence of the deletion in international samples suggests that the variant allele is globally dispersed. To obtain comprehensive prevalence parameters, randomised and wide scale testing is required.

In conclusion we presented a novel deletion of *ACSL5*, causing hereditary intestinal lipid malabsorption in the Australian Kelpie dog breed. *ACSL5* plays an important role in long chain fatty acid storage and metabolism. The improved health of affected individuals with age implies that genetic compensation of this gene beyond neonatal development is possible. This research identifies the first spontaneous animal model to validate key mouse knockout model findings previously reported. The AK model presents a unique opportunity to improve gaps in our understanding of *ACSL5*. A simple genetic test has been developed and validated to identify dogs harbouring the described variant. International testing of Australian Kelpies is warranted to obtain better estimates of global prevalence. At this time the disorder is presumed to be restricted to a single breed.

## Supplementary information

Supplementary Figures.

Supplementary Tables.

## Data Availability

The dataset used in the current study is available at Figshare https://doi.org/10.6084/m9.figshare.12380564.

## References

[1] Watkins PA, Ellis JM (2012). Peroxisomal acyl-CoA synthetases. Biochim. Biophys. Acta BBA Mol. Basis Disease.

[2] Watkins PA, Maiguel D, Jia Z, Pevsner J (2007). Evidence for 26 distinct acyl-coenzyme A synthetase genes in the human genome. J. Lipid Res..

[3] Mashek DG, Li LO, Coleman RA (2007). Long-chain acyl-CoA synthetases and fatty acid channeling. Future Lipidol..

[4] Arnott ER (2015). Strong selection for behavioural resilience in Australian stock working dogs identified by selective sweep analysis. Canine Genet. Epidemiol..

[5] Chew T, Willet CE, Haase B, Wade CM (2019). Genomic characterization of external morphology traits in kelpies does not support common ancestry with the Australian Dingo. Genes..

[6] Fyfe JC (2018). Inherited selective cobalamin malabsorption in Komondor dogs associated with a CUBN splice site variant. BMC Vet. Res..

[7] Fyfe JC (2013). An exon 53 frameshift mutation in CUBN abrogates cubam function and causes Imerslund-Gräsbeck syndrome in dogs. Mol. Genet. Metab..

[8] Fyfe JC, Hemker SL, Venta PJ, Stebbing B, Giger U (2014). Selective intestinal cobalamin malabsorption with proteinuria (Imerslund-Gräsbeck syndrome) in juvenile Beagles. J. Vet. Intern. Med..

[9] Gold AJ, Scott MA, Fyfe JC (2015). Failure to thrive and life-threatening complications due to inherited selective cobalamin malabsorption effectively managed in a juvenile Australian shepherd dog. Can. Vet. J..

[10] He Q (2005). Amnionless function is required for cubilin brush-border expression and intrinsic factor-cobalamin (vitamin B12) absorption in vivo. Blood.

[11] Kather S, Grützner N, Kook PH, Dengler F, Heilmann RM (2020). Review of cobalamin status and disorders of cobalamin metabolism in dogs. J. Vet. Intern. Med..

[12] Chang CC (2015). Second-generation PLINK: Rising to the challenge of larger and richer datasets. GigaScience..

[13] Kõressaar T (2018). Primer3_masker: Integrating masking of template sequence with primer design software. Bioinformatics.

[14] Koressaar T, Remm M (2007). Enhancements and modifications of primer design program Primer3. Bioinformatics.

[15] Untergasser A (2012). Primer3—New capabilities and interfaces. Nucleic Acids Res..

[16] Ewels P, Magnusson M, Lundin S, Käller M (2016). MultiQC: Summarize analysis results for multiple tools and samples in a single report. Bioinformatics.

[17] Dobin A (2013). STAR: Ultrafast universal RNA-seq aligner. Bioinformatics.

[18] Grabherr MG (2011). Full-length transcriptome assembly from RNA-Seq data without a reference genome. Nat. Biotechnol..

[19] Wang L, Wang S, Li W (2012). RSeQC: Quality control of RNA-seq experiments. Bioinformatics.

[20] Thorvaldsdóttir H, Robinson JT, Mesirov JP (2012). Integrative Genomics Viewer (IGV): High-performance genomics data visualization and exploration. Brief. Bioinform..

[21] Garrido-Martín D, Palumbo E, Guigó R, Breschi A (2018). ggsashimi: Sashimi plot revised for browser- and annotation-independent splicing visualization. PLoS Comput. Biol..

[22] Sewell AC, Haskins ME, Giger U (2007). Inherited metabolic disease in companion animals: Searching for nature’s mistakes. Vet. J..

[23] Koeberl DD, Pinto C, Brown T, Chen YT (2009). Gene therapy for inherited metabolic disorders in companion animals. ILAR J..

[24] Dandrieux JRS, Noble PJM, Halladay LJ, McLean L, German AJ (2013). Canine breed predispositions for marked hypocobalaminaemia or decreased folate concentration assessed by a laboratory survey. J. Small Anim. Pract..

[25] German AJ (2012). Exocrine pancreatic insufficiency in the dog: Breed associations, nutritional considerations, and long-term outcome. Topics Companion Animal Med..

[26] Jergens AE, Simpson KW (2012). Inflammatory bowel disease in veterinary medicine. Front. Biosci. (Elite Edition)..

[27] Bowman TA (2016). Acyl CoA synthetase 5 (ACSL5) ablation in mice increases energy expenditure and insulin sensitivity and delays fat absorption. Mol. Metab..

[28] Mashek DG, Li LO, Coleman RA (2006). Rat long-chain acyl-CoA synthetase mRNA, protein, and activity vary in tissue distribution and in response to diet. J. Lipid Res..

[29] Mashek DG, McKenzie MA, Van Horn CG, Coleman RA (2006). Rat long chain Acyl-CoA synthetase 5 increases fatty acid uptake and partitioning to cellular triacylglycerol in McArdle-RH7777 cells. J. Biol. Chem..

[30] Poppelreuther M (2012). The N-terminal region of acyl-CoA synthetase 3 is essential for both the localization on lipid droplets and the function in fatty acid uptake. J. Lipid Res..

[31] Teodoro BG (2017). Long-chain acyl-CoA synthetase 6 regulates lipid synthesis and mitochondrial oxidative capacity in human and rat skeletal muscle. J. Physiol..

[32] Wu M, Cao A, Dong B, Liu J (2011). Reduction of serum free fatty acids and triglycerides by liver-targeted expression of long chain acyl-CoA synthetase 3. Int. J. Mol. Med..

[33] Adamo KB (2007). Peroxisome proliferator-activated receptor γ 2 and Acyl-CoA synthetase 5 polymorphisms influence diet response. Obesity.

[34] Ellis JM (2010). Adipose acyl-CoA synthetase-1 directs fatty acids toward beta-oxidation and is required for cold thermogenesis. Cell Metab..

[35] Killion EA (2018). A role for long-chain acyl-CoA synthetase-4 (ACSL4) in diet-induced phospholipid remodeling and obesity-associated adipocyte dysfunction. Mol. Metab..

[36] Privette JD, Hickner RC, MacDonald KG, Pories WJ, Barakat HA (2003). Fatty acid oxidation by skeletal muscle homogenates from morbidly obese black and white American women. Metabolism.

[37] Rajkumar A (2016). Acyl-CoA synthetase long-chain 5 genotype is associated with body composition changes in response to lifestyle interventions in postmenopausal women with overweight and obesity: A genetic association study on cohorts Montréal-Ottawa New Emerging Team, and Complications Associated with Obesity. BMC Med. Genet..

[38] Rajkumar A (2018). ACSL5 genotype influence on fatty acid metabolism: A cellular, tissue, and whole-body study. Metabolism.

[39] Zhao C (2019). Adaptive evolution of the ACSL gene family in Carnivora. Genetica.

[40] Plassais J (2017). Analysis of large versus small dogs reveals three genes on the canine X chromosome associated with body weight, muscling and back fat thickness. PLoS Genet..

[41] Meller N, Morgan ME, Wong WP, Altemus JB, Sehayek E (2013). Targeting of Acyl-CoA synthetase 5 decreases jejunal fatty acid activation with no effect on dietary long-chain fatty acid absorption. Lipids Health Dis..

[42] Glick BS, Rothman JE (1987). Possible role for fatty acyl-coenzyme A in intracellular protein transport. Nature.

[43] Kang M-J (1997). A novel arachidonate-preferring acyl-CoA synthetase is present in steroidogenic cells of the rat adrenal, ovary, and testis. Proc. Natl. Acad. Sci..

[44] Oikawa E (1998). A novel Acyl-CoA synthetase, ACS5, expressed in intestinal epithelial cells and proliferating preadipocytes1. J. Biochem..

[45] Gassler N (2004). Expression of acyl-CoA synthetase 5 reflects the state of villus architecture in human small intestine. J. Pathol..

[46] Gassler N (2007). Regulation of enterocyte apoptosis by Acyl-CoA synthetase 5 splicing. Gastroenterology.

[47] Gassler N (2006). Molecular characterisation of non-absorptive and absorptive enterocytes in human small intestine. Gut.

[48] Bu SY, Mashek DG (2010). Hepatic long-chain acyl-CoA synthetase 5 mediates fatty acid channeling between anabolic and catabolic pathways. J. Lipid Res..

[49] Blaskovic S, Blanc M, van der Goot FG (2013). What does S-palmitoylation do to membrane proteins?. FEBS J..

[50] Spinelli M, Fusco S, Grassi C (2018). Nutrient-dependent changes of protein palmitoylation: Impact on nuclear enzymes and regulation of gene expression. Int. J. Mol. Sci..

[51] Sandoz PA (2018). The architecture of the endoplasmic reticulum is regulated by the reversible lipid modification of the shaping protein CLIMP-63. bioRxiv..

[52] Abrami L (2017). Identification and dynamics of the human ZDHHC16-ZDHHC6 palmitoylation cascade. Elife.

[53] Fairbank M, Huang K, El-Husseini A, Nabi IR (2012). RING finger palmitoylation of the endoplasmic reticulum Gp78 E3 ubiquitin ligase. FEBS Lett..

[54] Fredericks GJ (2014). Stable expression and function of the inositol 1,4,5-triphosphate receptor requires palmitoylation by a DHHC6/selenoprotein K complex. Proc. Natl. Acad. Sci..

[55] Lakkaraju AKK (2012). Palmitoylated calnexin is a key component of the ribosome–translocon complex. EMBO J..

[56] Senyilmaz, D. *et al.* Regulation of mitochondrial morphology and function by stearoylation of TFR1. *Nature* **525**, 124. 10.1038/nature14601. https://www.nature.com/articles/nature14601#supplementary-information (2015). 10.1038/nature14601 PMC4561519 26214738

[57] Blanc M (2015). SwissPalm: Protein palmitoylation database. F1000Res.

[58] Lo H-C, Yang R-B, Lee C-H (2014). Guanylyl cyclase-G modulates Jejunal apoptosis and inflammation in mice with intestinal ischemia and reperfusion. PLoS ONE.

[59] Young JM, Waters H, Dong C, Fülle H-J, Liman ER (2007). Degeneration of the olfactory guanylyl cyclase D Gene during PRIMATE evolution. PLoS ONE.

[60] Hoeppner MP (2014). An improved canine genome and a comprehensive catalogue of coding genes and non-coding transcripts. PLoS ONE.

[61] Goetzman ES (2017). Advances in the understanding and treatment of mitochondrial fatty acid oxidation disorders. Curr. Genet. Med. Rep..

[62] Spiekerkoetter U (2010). Current issues regarding treatment of mitochondrial fatty acid oxidation disorders. J. Inherit. Metab. Dis..

[63] Behrend AM (2012). Substrate oxidation and cardiac performance during exercise in disorders of long chain fatty acid oxidation. Mol. Genet. Metab..

[64] Gillingham MB, Scott B, Elliott D, Harding CO (2006). Metabolic control during exercise with and without medium-chain triglycerides (MCT) in children with long-chain 3-hydroxy acyl-CoA dehydrogenase (LCHAD) or trifunctional protein (TFP) deficiency. Mol. Genet. Metab..

[65] van Eerd DCD (2017). Management of an LCHADD patient during pregnancy and high intensity exercise. JIMD Reports, Vol. 32.

[66] Beynen AC, Kappert HJ, Lemmens AG, Van Dongen AM (2002). Plasma lipid concentrations, macronutrient digestibility and mineral absorption in dogs fed a dry food containing medium-chain triglycerides. J. Animal Physiol. Animal Nutr..

[67] Matulka RA, Thompson DVML, Burdock GA (2009). Lack of toxicity by medium chain triglycerides (MCT) in canines during a 90-day feeding study. Food Chem. Toxicol..

[68] Rutz GM, Steiner JM, Bauer JE, Williams DA (2004). Effects of exchange of dietary medium chain triglycerides for long-chain triglycerides on serum biochemical variables and subjectively assessed well-being of dogs with exocrine pancreatic insufficiency. Am. J. Vet. Res..

[69] Cotter R, Taylor CA, Johnson R, Rowe WB (1987). A metabolic comparison of a pure long-chain triglyceride lipid emulsion (LCT) and various medium-chain triglyceride (MCT)-LCT combination emulsions in dogs. Am. J. Clin. Nutr..

