# Supplementary material for: A large deletion on CFA28 omitting ACSL5 gene is associated with intestinal lipid malabsorption in the Australian Kelpie dog breed
Source: Sci Rep. 2020 Oct 26;10:18223. doi: 10.1038/s41598-020-75243-x (PMC7589484; doi:10.1038/s41598-020-75243-x)
Supplement: Supplementary file 1 — Supplementary Figures. [file 41598_2020_75243_MOESM1_ESM.docx]

**A large deletion on CFA28 omitting *ACSL5* gene is associated with intestinal lipid malabsorption in the Australian Kelpie dog breed**

Mitchell J. O’Brien^1*^, Niek J. Beijerink^2,3^, Mandy Sansom^#a^, Sarah W. Thornton^2,#b^, Tracy Chew^4^, Claire M. Wade^1*^

^1^ School of Life and Environmental Sciences, Faculty of Science, University of Sydney, Camperdown, NSW, 2006, Australia

^2^ Sydney School of Veterinary Science, Faculty of Science, University of Sydney, Camperdown, NSW, 2006, Australia

^3^ Veterinaire Specialisten Vught, Reutsedijk 8a, 5264 PC, Vught, The Netherlands

^4^ Sydney Informatic Hub, University of Sydney, Camperdown, NSW, 2006, Australia

^#a^ Current Address: Callicoma Kelpies, Grafton, NSW, 2460, Australia

^#b^ Current Address: 1416 Fairway Drive, Los Altos, CA 94024, USA

*Corresponding author(s):

Email: [Mitchell.obrien@sydney.edu.au](mailto:Mitchell.obrien@sydney.edu.au) (MJO), [Claire.Wade@sydney.edu.au](mailto:Claire.Wade@sydney.edu.au) (CMW)

**
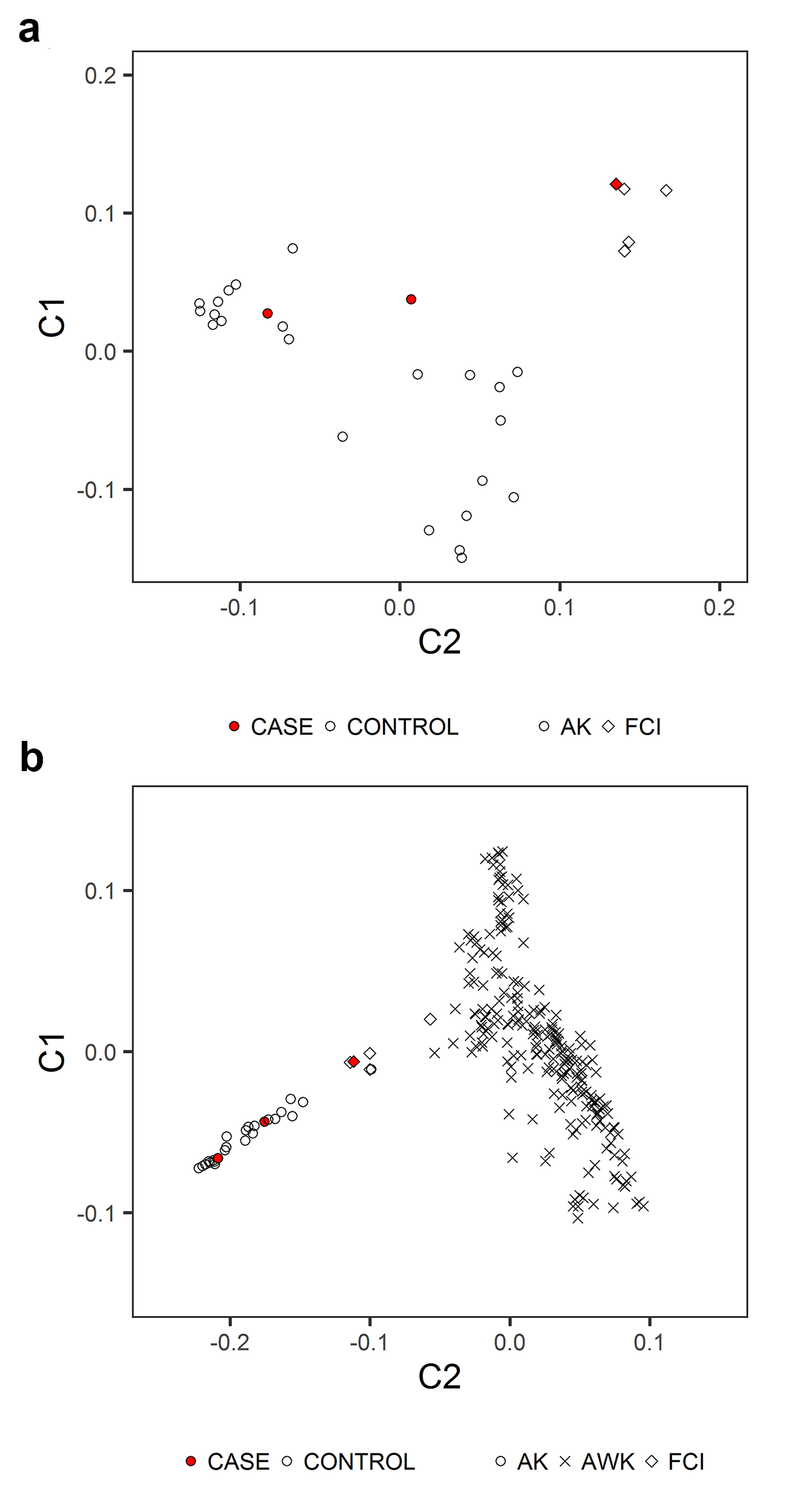
**

**Figure S1. Multidimensional scaling plot of genotyped Kelpies.**

MDS plot shows the distribution of case/control samples included in the GWAS. Kelpie registries differ in selective choices and geographical origins, samples have been labelled according to their respective registry. AK = Australian Kelpie; Australian population selected according to a conformation breed standard. AWK = Australian Working Kelpie; Australian Kelpie selected primarily for strong working ability. FIC = Federation Cytological International; International population allowing co-mingling of Australian populations.  **(a)** dogs used in the primary association (n=30) **(b)** dogs included in the validation association (n=255).


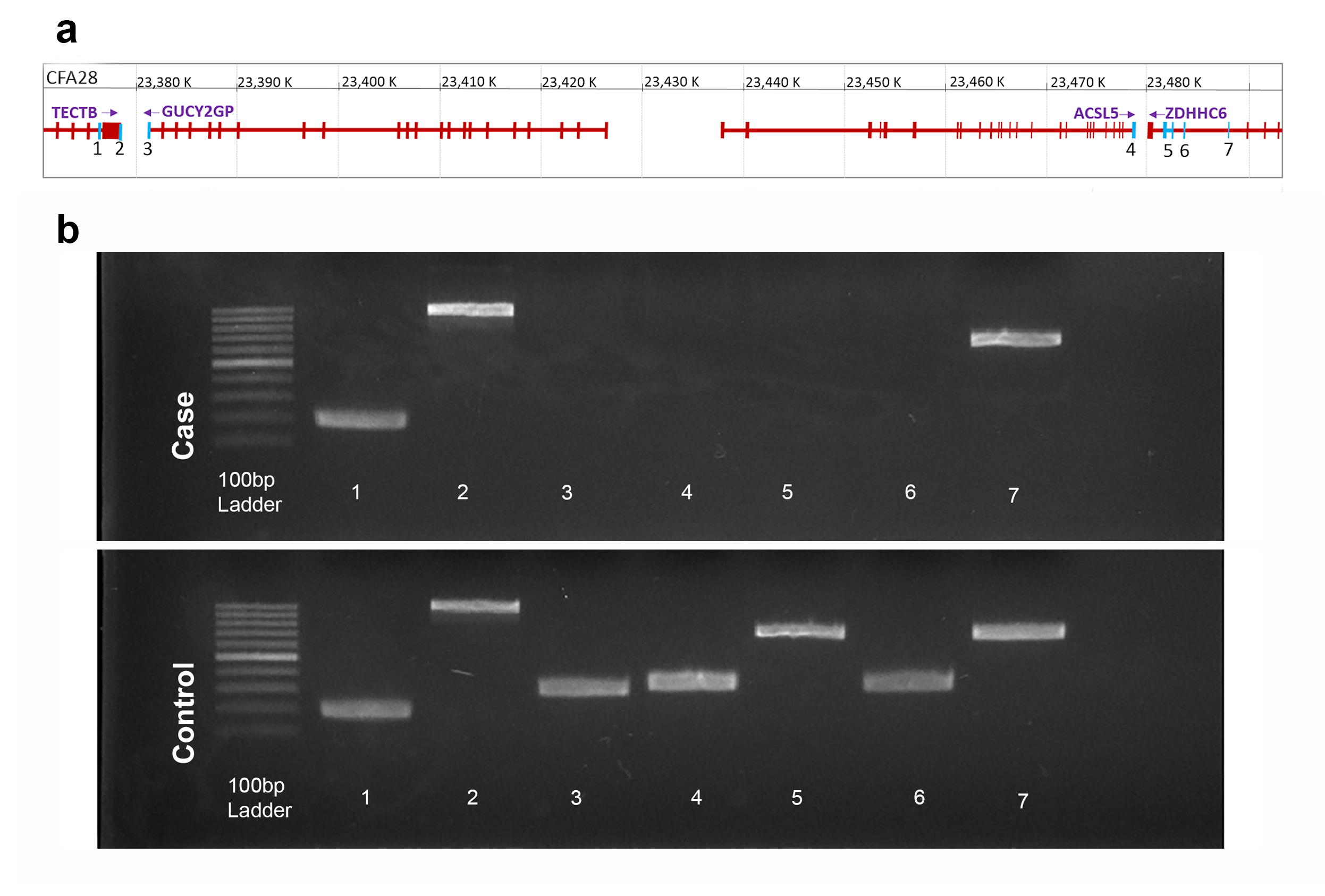


**Figure S2. Confirmation of deletion through PCR.**

**(a)** region on CFA28 (28:23370822-23493334), flanking a series of uncalled SNPs on canineHD BeadChip. Gene predictions are represented in red. Blocks are exons and untranslated regions, connected by introns (horizontal lines). Gene Symbols and arrows showing the direction of transcription are indicated in purple. 7 primer pairs were designed to amplify the edges of the suspected deletion. The regions amplified by the primers are coloured in blue. **(b)** PCR results. Lane numbers correspond to primer pairs illustrated in supplementary figure 1a, primer details outlines in Supplementary table 1. The top and bottom results come from a case and control respectively. In the control sample DNA amplification was consistently seen with all primer pairs, while it was only possible with primer pairs 1, 2 and 7 in cases. These results were indicative of a deletion between 101.6 kb and 105.2 kb in affected Kelpies. Gel image has been cropped to improve clarity and conciseness. Full gel image can be seen in the supplementary figure 3b.


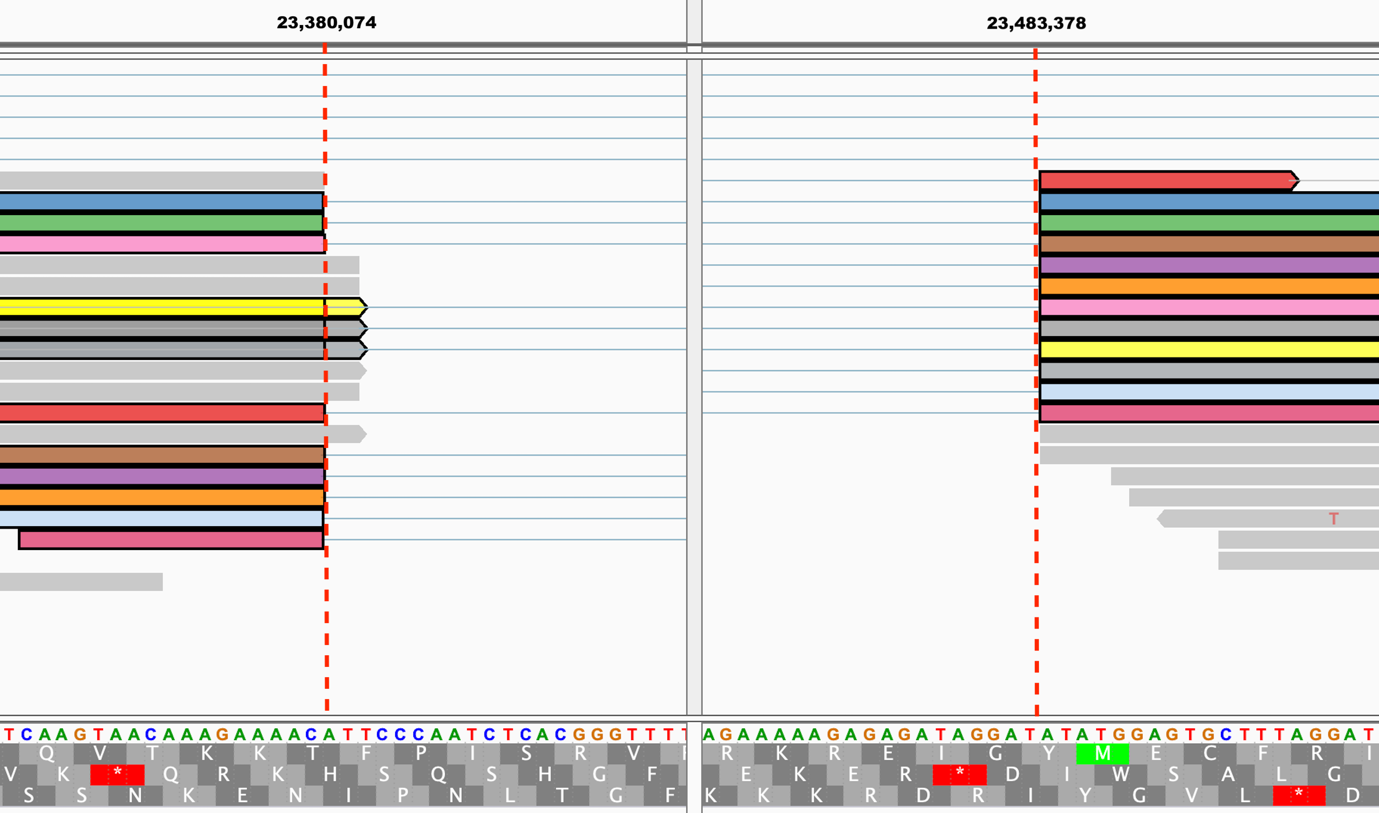


**Figure S3. DNA reads from an affect Australian Kelpie mapped to CanFam3.1 captured a 103.3 kb deletion (NC_006610.3CFA28:g.23,380,074_23,483,377del). Positions are marked by dashed red lines. Paired reads flanking the gap are coloured the same. Visualised in IGV.**


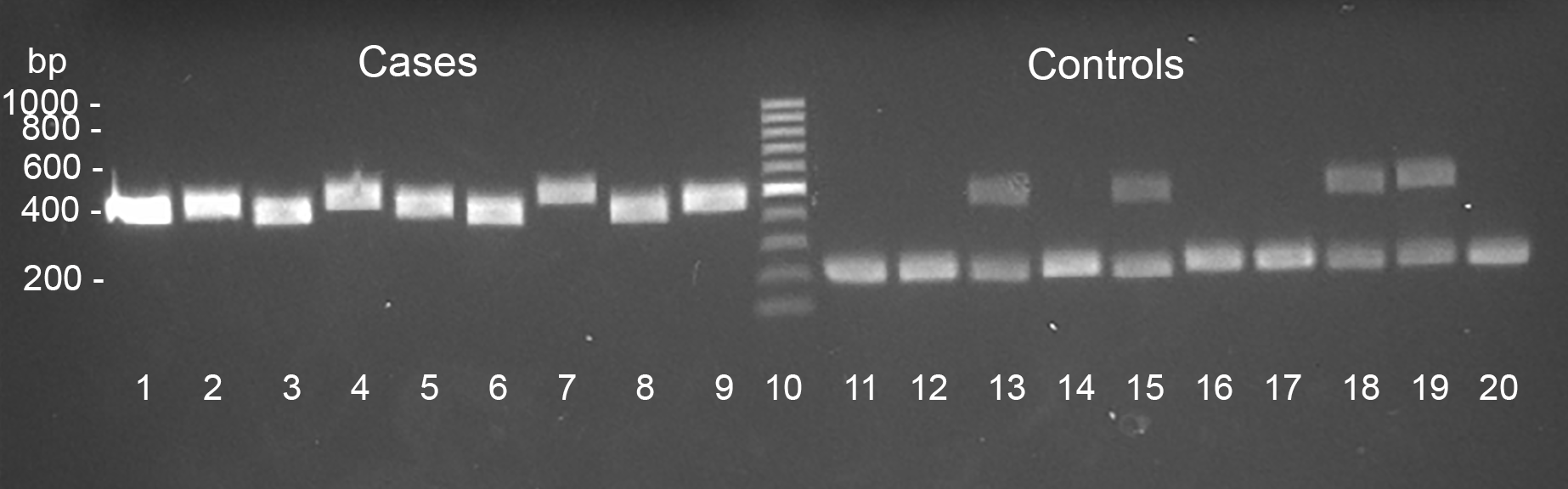


**Figure S4.** **Multiplex-PCR diagnostic test results for 19 Kelpie samples.**

Lanes 1–9 include case samples, each tested homozygous for the mutant allele. Lanes 11-20 include controls. All control samples with the exception of lanes 13, 15, 18 and 19 were homozygous wild-type while the others are carriers for the deletion. Lane 10 is the ladder (100 bp). All samples included were AK or international Kelpies. The trait has not been identified in AWK to date. Gel images have been cropped to improve the clarity and conciseness. Original Gel image can be seen in the supplementary figure 3a.


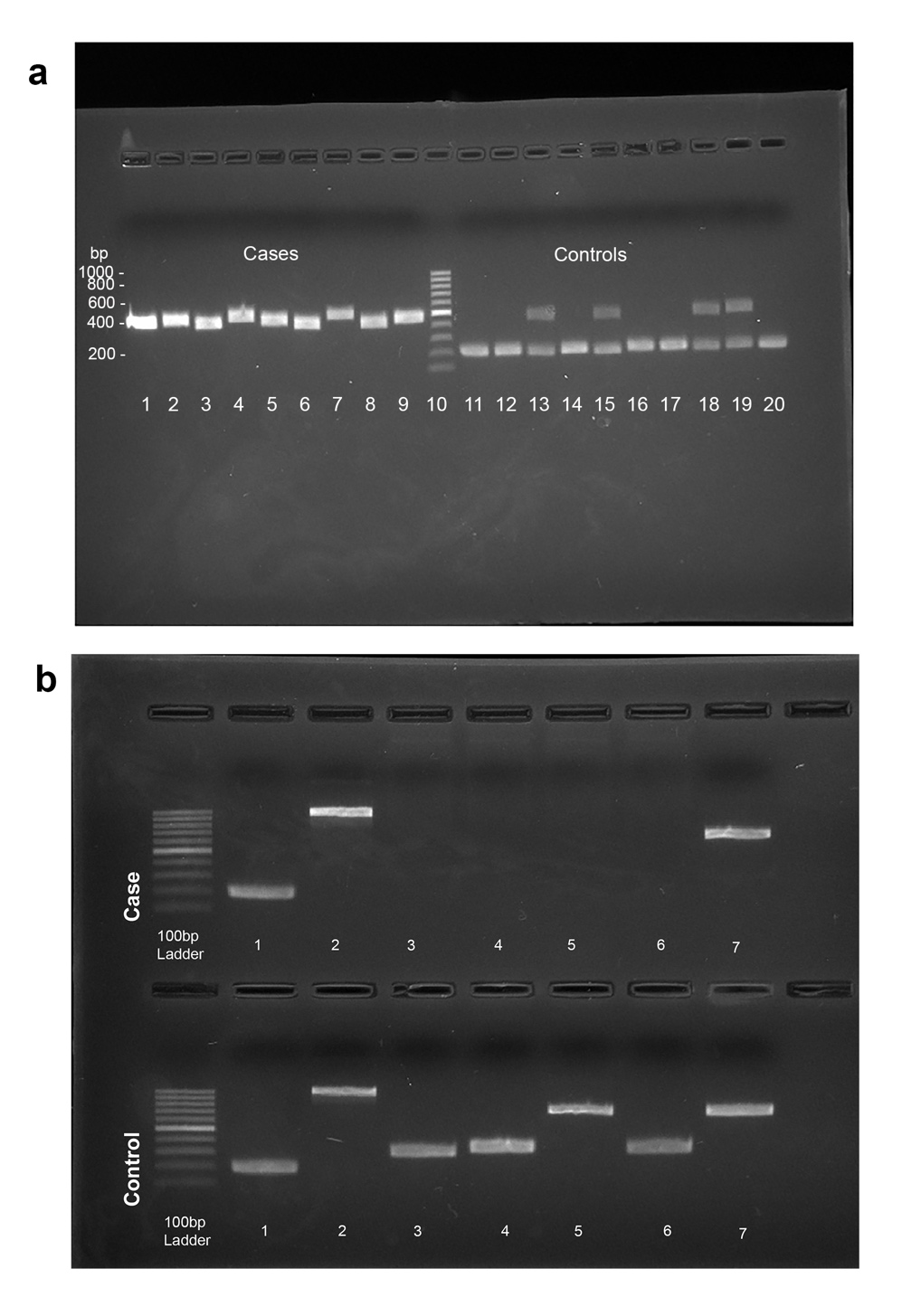


**Figure S5.** **Full size, unedited PCR gel images.**

(a) full scale image of PCR gel seen in figure 5. (b). full scale gel image from PCR gel included in supplementary figure S1b.

>AK_ZDHHC6_prediction_Trinity_denovo_alignment

GCTCCGCGCTGCAGCCCCGGACAACCGAGATCTGCTTTACTGCTTGCGCGGGCCTGACTT

TCCCGTGACGGGGTTGTCGGCGGAGGAGAAGACCCGTTCCGGGTGAGCCCAGGCCGCCCC

GGAAATGCGATGGCCGAGGAGCGGGTACCGAGGACCAGGCTGAGAGGTTGGGTTTCGAGC

CAAGACCCTGGATTCTCCTAGTTCACATAAAAGAGCTCTGAGGGTGTACCCGTGAAAATG

GCGTGATCTGCATTGGCCTCCCACAATTTGAAGGCATGGCAGTTAAGGAACACACGTGGA

CTCGTGGCACATGGAAATGTGTGCACAGAAAAAGGAAATCTATGATTTTCTAAAAGTAGG

AAGGCATTCTTCCTCACCAAAATGGATACATTCTGCTCAGTTATCAAGTTTGAAAATCTC

CAAGAACTAAAGAGACTGTGTCACTGGGGTCCCATCATAGCCCTCGGTGTTATAGCAATA

TGTTCCACAATGGCCATGATTGACTCTGTGTTGTGGTATTGGCCTTTACATACAACTGGA

GGAAGCGTGAATTTCATTATGTTGATAAACTGGACTGTCATGATTCTTTATAACTACTTC

AATGCCATGTTTATTGGTCCTGGCTTTGTCCCTTTGGGGTGGAAACCGGAAAATTCTCAG

GATAGCATGTACCTCCAGTATTGTAAAGTCTGCCAAGCATACAAGGCACCACGGTCACAT

CACTGCAGAAAGTGTAACAGATGTGTGATGAAGATGGACCATCACTGTCCTTGGATCAAC

AACTGTTGTGGTTACCAAAATCATGCTTCGTTCACACTGTTTCTCCTTTTAGCACCACTG

GGTTGTATTCATGCTGCCTTCATTTTTGTTATGACTATGTATACACAGCTTTATAATCGG

CTCTCCTTTGGGTGGAACACGGTAAAGATTGATATGAGTGCAGCCCGGAGAGATCCCCTT

CCAATTATTCCCTTTGGATTAGCAGCATTTGCGGCCACCTTGTTTGCCTTGGGATTAGCT

TTAGGAACAACCATAGCTGTTGGGATGTTGTTTTTTATCCAGATGAAAATAATTCTCAGA

AACAAAACTTCTATTGAATCATGGATTGAAGAAAAGGCTAAGGATCGAATTCAATATTAC

CAACTAGATGAAGTCTTTGTTTTTCCCTATGATATGGGAAGTCGATGGAAGAACTTTAAA

CAGGTATTCACATGGTCAGGGGTCCCCGAAGGAGATGGACTAGCATGGCCAGTAAGAGAA

GACTGTCACCAGTACAGCTTAACAGCACACAGTGAGAGAGAGAGAGGCAGAGACACAGGC

AGAGGGAGAAGCAGGCTCCATGCACCAGGAGCCCGACGTGGGATTCGATCCCGGGTCTCC

AGGATCGCGCCCTGGGCCAAAGGCAGGCGCTAAACCGCTGCGCCACCCAGGGATCCCTGT

TAGTGACAACTTATCTCAGCCCAGTGCAGCAGGATGAGAACCAACAGCTTAATGAATGTC

ATCAGCTCATGTCTTCATTTACCTGGCTCAACCCATCTTCTTCAGCTATGGAGATCAGCA

CAATGAAGAAAAAGTCACTCGAGACCATAGTCTTGTTGGCTTCTGTCCAATGGGTGGCCA

GAGGGCCAGAAGCCAAGGACTCTTGGTGAGTGCAGGAGCTTATTGAGCTTTGGCCTCATA

TCCAATCAGGTGAGTCAATTGAGCAAGAGAAGAACCAGGACAGCCAATGGCTGAGCTCCA

GGATGGAGAGATGCCAGAGGAGAAGGTAGAGCATGAGAGAGAGAGAGAGTGTACTCCAGG

TTAGTTGGAAAGAGTTGAACAGAGAAACCATTGAGCTCTTTTATTCTCCTTCATTCTCCA

AACTAATTTTCCCTAAGAAGCAAAATTTCAGGTTTAATGGACTGTCCCTTACAAAAATCA

CATTTATGAAAACACCGTGAAGTTATTTTTCCATGAGGAGACATTAGAAAAAGGTCCACA

GGAAGCAAAGGAAGGAGACACTGTGTGGGTTAGGAAAGATCATAGATGTTTACACATAAA

GAAATTTGGAGGTTGTCTGGGCCACCATTTGCCTTTACTGATGTGAAGAGGCCAAGAGAA

GAAGAGTGGTGTGCCCAAGGTTGCATGGGTACAGGGTATAACCTGGCTTCTTTTCACCAG

GCTTGGTGATCTTTTCCTATATTCTTTTTGTGTCTCCTTTTATATTTCTAATAGAAAATG

ACTGTCTATTCCCCTCAACCTCTGGGTTCTGACTCCTGTGCTCTGCAAGGCCCTTCTCCA

TTCCTCTCCCATCATTGGCCCATGAATGACCTTCTTTTTAGGGTCAGACCACCTGGTTGC

AGAAGAATGCATATGACCATACTGGGAATGTGCTGATGCTCAGCAATGTCAGCTGTCTGT

CTTCTTCAGCAACCTCTGGCTTCTTGGGTAGAAAAAGCTATCAGGAAGCAACTAGAAACC

CAAACTAGATGCTTTGTATGTACTAAACTAAATTGCAAACTGCTTGAAGGCTAGGACTTT

CCTTAGTCTCTCCTGTAAATCCAGGGCTTAATATATAATTTGGCACATCATAGGTGTTCA

ATAAGAATTGTTGAAAAGAAATGAATGAGTGTCCACAGGAAAAATGGAATGATCTTCAGA

GTAGTCAGAGGAGGTTCCCAGAGTAGGTGTAGCCTAAGCCAGGCTCCCAAAACATGGCTA

CTGGTAATTCCTTTATTTATTTATTAAATGTATTTAAAGAAGCATTTATTGGGCCAGGCA

TTCTTCTAAGTGGGGAAGGAATTGCCATTGTTTATGTAGAGGTTAGTTAAGGCTTCATTT

ATGAAGAGAGAAGTGTGTCCTGTTCTATGAGAGATCACAGTGCCGGGATCCTACTAAGAA

TCTTCCTGCCCCCAACCCCTTGCTCCCTAGAACATTCTTTTTATTTTATTTCCCCCCATA

GACATCCTGGTAGTGAATTGGGCTGGCTGGCTGACACACCTGGGAGGGTGAAATTGTGGA

ATGCAAAGGTGTGGCAGTGACACCTGGGCGACTCAGTGTCCTGAAGCTTTGAACCTCAGG

CCCCAACTTTCCCTGGAGTCTTTTCTCCAGGAGCCAGTGGCCTTCAGGTGGCTTATCCAA

TGTGGGGCTCTCCTCCAGGATTTTGAAATTGTGGGCAAATTTGTGCCTGTGTAGCAGGAA

CCTGGATTCTGCATTCTAGATTAGAAGCTTAAATCTGTTCTAGGGCAGCATCCACCTGTT

CTGGGAGATATCCCTGGGGACTTCCCTAGGAGGCAGCTGTGAAGTTGGCAAAGTCATCCT

AAGGCACATGCTCCAACCTAAGCTTATAAATATTCTGCTTGATGACACAGAGGACTATGT

GCATTCTGTGGAAATGATAATATTAACTAGTGTGAGGCTGTCATTTGGATATAAATAAAG

AGGTGACCCTGGAGAGTGGGAAAGTTGGGGTGACCACAGTACATGTGCCTTTCCCATGGA

GAAGATCCATGGATATCATCAGATTCTAAAATGGGTGGTTGATCCCAAATGGTGGAGTTG

ACCTATCCAGTGTGTATAAGGGAAGTTGGCTTCTTAGGGGCAGAGATGTACATTGTCAGT

CCTGTAAAGGAAAAATCAATCTCCCATGTGTCTCCACTCTCAAAACTCATGACAACACCT

CTTCACTTCTGACACACTTCTGTCCCACACATCAATTTGGTGACACCAGCTAGATGTCCT

ACAATGTAACTCTGTTCTGATACTCCCTGGCATTATTGTCAGATCCCACTGGTGAAGGGC

GCAGTTCCACAACACTACCTACTTCAGACACCAATCCCGAATTCAAATTATCACCTCTAT

TTCTGACTGAATGGCTATAAATTAGAGGTTCCCATGACTCCCTCATCTGGTTCAATCAAT

TTGCTAAAGTGGCTCACAGAATTCAGGAAAAACAGTTTACTTACTAGATTATAGATTTAT

TATAAAAGATACAACTCAGAAAGAGCCAAGTGAAAGAGATGTTTAGGGCAAGGTGTGCGA

GAAGGGGCGAGAAACTTCCATACCTTCTCTGAGTCTACTGTTCTACCAGCACCTCGGTGA

GATCACCAACCTGGAAGCTCACCCAACCCAGTCCTGTGTTTCTGTGGAAGCCTTATTACA

TAGGCATGATTGATTAAACCATTAGCCATTGGTGATTGAACTTGAATCTCCAGCCTCTCC

TCTCCCCAACCCCCCAACACCCTGGTCTGATTGCAAGGAGGAACTAAAGTTTCAACCAAC

TAATGGTAGCTCTCCCAGGCAACCAGTCCACCTCCTAGGGGCTTTCCCCCCAAATACATC

TGCGGTGTGGTTGAAAAGGGCTTATTAGGATTAATGAAAGACAACTTTATCTTTCATTGC

CTTTTTGCTTCAGAAACTTCAAGGGTTTTAGGAACTCTGTGCCAGAAATGGGGATGAAGA

CCAAAACATACATTTCTTATAAATCACAGTATCACAAATCCTGTGTAGTTGTATATTTTA

CAGAAGGCTTTGATCTTTCTGCTGAACAAGTTGAGAGAAAATGTCAAGGTTGTCCTCCCA

GAACGTAATGACAAAGCAGCTTCAAGGAGAGGCCAGTCAAAGGAGCACCTGGTGGTGCCC

ATGTGGGCTCTGCTTTGGACAGACCCTTGCTGAGAGGGCATCTAAGACAGCTGGCAACCC

TGTTCTAGCACCTGTGTGGTTCTGCAGGGGAAAGGTGCAGACCTGTCTCATGCCTCCAGC

CCAGGGCCAGAGAGCCCAGCCCATCTCCTGCCAGTGGAAAAATAGACATTTGTCTGTGCA

AACTAAGATGGTGAAGCCCTACTTTCCATGAGATAGGAATAGGGCAATAGAAAATTCTGA

CCCAGTTGTTCCCGCTGCAGAAAATCATGATCATTTTGTATGCAATGATTTAATGTATTA

TTATTTAAATATTGTTTCAAATGGCTGGTTTTTAGCCCAGTACCGATACATTTTGCTTCG

AAGAAGGGCAGTACACATTTTAAAACACTTTTATATATGTTGTCATGAAACACTGGCCTC

AGTTCTCTCATTTAGGAGTCGTGGGGACTGAACAAGAGCTAGGGAAATGATAGACATGCC

AGCTCAGCTTGATCTACAGCCCTGTGTTAAGAAGGATTCTGAGGCCACTGGTGAGTGCTA

TAGGAAGATGCTTCCTGATTGGTCAGCTCTGCCAGGAGTATAGGAGGAAGGGTGTTGGTA

CGTTAAGCCTCATAATGTTTATTATCTTTTCCAACCATGAGATTCCAGGACTCTAGGGTA

AGGGTCAGCAAATTGCAGCCCTTGGACCCAATGCAGTCCACTACAAGTTTTCATTTAAAT

AAAATTTTGTTGAAACACCATTGCACTGCTTTTTCACACTATCTGTGGCAGCTTTTGCCT

ACATGGCAGCTGAGTAGCTGTGACAGACACCATAAGGCCCACAAAGCCTAAAATATTTAC

TATTTAGCTCTTTACAGATAGAGCTTGTCAATTTTGTTTTACGGTACTTAAGACACGTTT

TAAAAAGGTGTACATCCAGGGAAGTAAGTGTTAACAATGATTAAGTAAGTCAGTTCATTT

GATACAGACAACATTTATTTAACACTTCATAAAGCAGGCAGTCTCTGTTCCCAAGGGGCT

GGAGAACTGCACAATGGTGTAGAAGGGTGAAGAATAAGGTATAAGGAAGGGACAAATAGA

AAATGAATAAAGAACAAGAAAGTAAGTGTAGAGCCCAGAGGTAACTTCACACTTGGGGAT

TGGCTGAGTGGATATACTACATTTCTGGCTGAGCAGAGCATTTACAGGGAAATGAACTTT

ATTGAAGATTTGGTTTCCTGCTGTGGCACCTGGGAACATCTCCAACTGGGGCCTAGAAAT

GTCTTTCAACCTTAGGAAGACCTTAGTAGCTTTATCAAGCAAGTGAGGCACCCCGAAGGA

GAGGACCTTGCTCAATATCATAATGCCATGTACAACTACCTAGGTGTCCCATGGGGCGCT

GATTCATTCCACTGCAACTCGTGGGCTTCACAGAAGAGAACATCTTCATTCAGGGCAGAA

TTTATTTAATTGGTTCTAGGGTAAGAATTTCACACCATGTTTTCAAATGTAACCTGGGAA

GCCAATGATTTTTTGTTTGTTTATTTATGTGCAAGAAAGGGAGCATGGAAGTCCCTGCCT

ATTGGTGGAGTTCAGGCACTTTGGTTGTTCAACAGTTTGGGTTCTCCAAGAAGTAGGTAC

TGAAACAGGAGTAGGCAGGCAAGAGATTTATTGAGGGAAATGCTTTTGAAGGATACAGAG

GGAGAGGGCAGAAGCAGGTAGGCCTTGGACCATAATGTTGGTCTGAGCCCTGTGAAGGGA

GAGAAAGGGAGGAAAGAAGAGCCTCAGACCCCATGGCAGCTCCAAGAATGTCTTATCTGG

GCTGATGGAGCAACGTTGCATGGGCAGAAAAAGATTGGCTCAGACCCCTATCATGCCCAG

CCATTGATCAACAGGAACCTATGGAAATGTGGCCTTGGCTGAAATGTGTAGTGGATTTTT

AGGGGCAGCATCTAGAGGATGTTTGCCAACTATGCTCCTGTAGCAGGTTCTCTTGAAGAG

GACGCTGGGGAGTACATCTCTATGATGCTACAGCATGCATTGTTTGCCTGCACACCCTTG

CCAGGGTCTGTGCCAGGCCTGGAAGCTATGAGGATCTAAGGAACATTGTTCCCATCACCA

GGGAGCTCATGATCTAACCAGGAAGATCAATTTGCACACCTGGACAAGGGAATGTGCATC

AAATCATGGCACAGGCAATAATAAAAGGAGAATGACAGCAGCCTGAGATTGGCAGGAACA

ATGTGTGGTAGTGGTTCAAAGAGGCATCCTTGGAGGAGAGCTTGGAATGTTCTCATTAGG

CATTACGACGGGCATGTGTGCTGGGTGTTCTGCATCATTTTCAGCCTTGCCCACGCCCAC

GGGTTGCCTCCACCTATTCAGCATTCACTGAGTATCTAACCAAGTACTAGGCTCCATGCC

AGCTACTGTGATTATGTGGGTCCCTGGTCACAAACAAGCTCATGATATAGAGGGGCCTCA

CAGACACGGATGAGTCATTCAAAAAAAAAATCTACGGAAATATGTGTGGGTTTGTGTGGG

ATTCCTAATGACTGGAATCCAGCTACTATTCACAGTGAGACACAAACAATGGGCAATTTC

TGGTTTCTGTTCCTAGGGCTTCCAGGGTGGTGCCCGGAGATCTGTGTGGTTATGATGTAC

CTGGAGACTTCCGATAAAAGAGTCCTCTGACCACACTTTAATGACTCTAGGGAGGCAAGA

GAGAGAAACCCACAAGCCACTTGGAGGTTTGGCTGTTACAGTCTATTTAGAAACTTTGGT

AACTGTAAAAAAGTATTAGCAAGTAAATATATGTGAATATGTATTTTGTTTACGTTATGG

GGCAAAAACTGGCAGCCATAGGCTGAATTATCTTGAAAATCTGTGGAGATCTTTTTGGCC

TGAACAGTTGCCAAATTCTAAAAAAAATTGGAGGTTTCCATAAAAGTCATCTTTCCTGTC

TCTCATGAAGATAAAGAGCCCTGACAACACAGCCTACATTTTCCATGCAATGTTTGGCTG

GAGATGGTAGATGCCCTTAGATGGGTTATGTACGCCCCATTTGACGTCAGCCCCCGCTAC

TCCCTATTAATTACACCTAACCCATTTTTCTCATTTATATTACCTGCCTGGTGCTAAGAT

GCTTTTGGATTTGTGATACATTGGACAGACTCCCCAATTCAATTGGCAAGTGTGTTTGTC

TCTGTGTGGTTCTGCCCTTAAGTAGTGCAGACTGAGCCCAAATGGAAAAGGGTTTTTATC

TGGCCACGACACAGCCATCAATGGTATGGATCACTGGGCACCTCCATTAACAGAAGTTTG

CACAGATCTTCTCTGGGTTGAAAGACTCATCCCAGGACCAGGTACTCAGAAAATGCCTGC

AGTAGGAAGATTTACCTGGTCACAGGAGCCAATTTCTCTACTTAAGACTATCATCAACCA

ACTATTTAAGGACAAACTTTGACATAGCCTTAAGGAAATTAAAGCTAAAGCTACTATTCT

CTGAAGGCAACAGAACTCATAGAGTCAAAAGCAAGGAAACACACACGCAGATATGTACAT

AGATATTCTCACAGATATGTATGATATGGATCTAGATGCACATATGTATTAACATACACA

TGATTTGGGGAATGCCGGAGAAATCACTGAACGGAGAATGGGAATAAGCGGTAGTTTGTG

AGAAACAGAAAGGAAATGAATCTAAAAACTCTCTGTGTTTTAAAGGTTTATTTATTTATT

TAAGAGAGAGAGCGCGCACATGTGCACACGTGCGAGTGCGTGAATGGGCAGAGAGGCAGA

GGGAGAGAATCTTCAAGCAGACTCCCCAGTGGTCATGGAGCCTAACTTGGGCTCAATCTC

ACAACCCATGAGATCACAACCTGAGCCGACACTGAGAGTCAGACACCTAACTGATTGAGC

CGCCCAGGTGCCCCTAAGAATAGGAATAAGAGAATAAGAATTCTCTTATGAGTGCTAACA

TGAAATCACAGGAAGATAGGTTTTGGAGTCAGATGGCCTCAGGTTTAAGTCCCAACTGCA

CCACATGCTAACTGTGTGACCTCAAGCATTTTAGTTAACTTCTCTGAGCTCTACTATTTC

ATTCTCTGAAATGGGTGTACATATTCCTACCCTTTGGGTTTTCATGGGCCTGGCCTATGG

TAGGTGCCAAAAGATATTGTTTCTTTTCACCAACTTGAACCTACTGCCTTGTTTTGCACC

CCGATGCCCAATACTCTCTGGGACTCTGAAGTGGGAGCAGATCTAAAGATACCCTTTCCT

TTTCCATAAGTGGATCTTGTAGCTAGCTCAGAGATATGAGGGACCTGGTACTAGCTTGGG

ACTGCTTCGTATTCTTAGAGCATTTTCTAATCAACTTTTTCTGGTTTTCCTGGTGCATAC

TGTATTCTGCGAGTAATGGGCCCAAGAAAACCCATGCAGGTGGCATGATCTGGATGCAAT

GGGTCAAGATCCAGGAGCCCATGAGATACCTGGCTGGTTCCAGACTTCTGGTACTTTCAT

ACCCCTCTGCCCACTCCATGATTCCTACTCTCAGGTAAGGAGCACTTTGCACCGGAATCT

CCACTTCAGAGGTGAGCTGTGGCCATGCCCATCACTTGTGCTCTCCTGGCCCACGAGACC

GCCCTTCCCCCATGGGGAGTTTTGCCCTCCTGCTCCCAGGAGGATCCCTCATGGGTGGTG

CTCACCAGGTCTGTGCTCTTTGGTTGGGGCTGGACCGGTGATAAGAAGCTGGGTGGGCTC

TGTACTCTAATACCACACTTGCTATCCCTGGTTAATGTTTTTCTCACATCATTCCACCTG

GAGCATCTGTTTGTATTGTTTATCAAACCTTAGGGTTTGGATGAAACATTGTTGGCCATG

GGACAAGCTGGGAACATGGCCAGCCTTTGAACAGGACTCTCCCCACATTCTGTTACCCTT

GGCTCTTCTCTTTATAGCCATCTTTCACTGCATCTTCAGACTTTCATGTTCCCTAATTCC

CTGTGATGCCCAGTACCTTCCTCACCTTGGTCTTCCCTCACCTGCTGTGCCTCCTATGGG

TTACCCTTCCTCCCACCTGAACATCCGAGACCTCAACTGCTCTCAGTTAAATGTTAGCAA

TAGAGCAAGCTGATCTAGCCCGTGTTTGGGAATCACATAACTGCCTTTCAATTGTTAATG

TGTGGTTTACTAGGTTTGTGTCCTTAGAAAAAGTATGTAATCTTTCTGGGCTGAATGTAC

ACATTTTTAAAATGTGAATGCTAACCTCTATTTGTCGGTCTAACTGCCATTTTCTCCATA

AATAATAGTGAAGTTCTTTTATTTCTTTCTTCTTCTTTCTCGTTGAACTACACATCCTGG

AAGCCAAGGCTGAGTCTGCAAGAGAAGATTAAAGATCTGTTTTGTGTGTGACGAGCTGAA

GTAGAAAAAATCATAGGACTTAATTCTGGCTTCAATTTTCAGATGGGTGATTTTTGTTGG

AGCCCTACAGGGAACCCACTGACTTGAGTCCACTTCTCGTCCATCGTTACTGGTTTTCCG

CTGCTCCCTTTGGACAGGCCCAAACCGTCTGTGACAAGTTGCCTTCACTGGCCCCTTCCT

TTGGCCTGACTGCACAAAGGACTCTAATGAACTTCTGCCTCCTGTGCTGTAACCGTTTCA

TGAGGGCAGGGGACTCACCTTGGTGCTCCTTCCCAAGCCCACTCCGCCATCCCCACCCCA

GGGATTACCTCAGTCCCTGATACAGTAGAAGCAAAATAAATCTGTTGAACAAATGGTTGA

TGGAGAGGTTGGAGTGGGGATAAGGGGAAATGACTGCTACTGGGAAGGGGTTTCTTTCTG

ATGTCACTTGAAATGTCCTAAAATTGATTCTGCTGATGGCTGTTATTCGCCTAACAAAGG

GGTTCTGAGATTCCTTCATAGAAGAGTTCTACTGTATCAATACCATTATCCATTCTCAGG

CCTGTTTAGCTGCCAACGCCCACCCTCCTCATGATGCTCCTGTGGCATCATCTCACTGAC

AGTGTGAGGGGGAGAGTGATTGGGAAGGATGAGGGATATAGAGAAGTTAAAGTCACTGCC

AAGGAAGGACCCAAGGAAAGGCATGTAAAGCATCTCCAACATGTGAGAACAAGTCACTAG

GGTCTCCATGACTTCTTCAGGGTCGCAGTCAATAAGTTGTTCATCTGGTGCCAAATACAC

TCACCCCTACTCCCCTACCCTCTCACGACCCAGCGCTGCTCAGCCCCACTCAGGCTTGGT

TTCTTAGGAGCAGTGAGAGCTCTCTTGGCATCATGGAAGCATCCAGACTGGGAGCTGAGT

GCCCCCACGTGATTTCTCAGGATACTTAGCTGCAGCCCCTGGGTCGGGCTCCTCTACTGA

GAATGAGGCTCCTGAGCTCAAAGCTTCACTCCTAAGTCAGAAAATCCCCACCAGGCAAAG

CCAAGAAGACACCACTTGCAGCATCATTAAATTATAACGAGGCCTGAAACTCACTCCAGA

AGCCTGGGTCTGTTTGTCCTTGCGGTCGGAAGGAAGCAGGTGATTGGCTGGCCGAGCTCG

GCCTAGCAGGCCTTATCTCTGGCTGGTTATTATTTGATTAAAGAGGCTTCCTCCTCCTGT

GGTGTTTCAAAACTGTGCTGGGAGAGGTTTGCTGCTGCCTGGGGAGTTTTTCTGAGGTTA

CTCCCGAGCACCCAAAGTCCTTCGGTCTGCAGCCATACAGACACCCAGAGAAGCTACAGA

CAGGCAGAGGCTATTTTCTGATGAGTCTTCATCAGCTTTGCTAATCAGCTGGTTGAAAAT

AATCCCTCAAACACTACAGAGTCAAGGATACAGGTATTACAGGTAACTTTTTTTTTTTTT

GCGGGGGGAGGTCTAATTTCGTAGAATGGCATGATATTTGAAATGGGGGGTAGGGTGGAG

CTTTGGGCCCCAGAGTGGAAAACAGGAGAGGCAGAGAAAGTGGAAAAAGAAAGTGCAAGA

AAAAAAGACTCAGAATCTTGGAATTCTACAGAAAGGCAAAGCCAAGATGTGAGTTATTTG

CAGCTGGAAAGAGGTGTGAGCATGATTGCATCACTGGCATTTGAGTGTTTTGTTCCCTAT

GAAGTCCTTCCTGGAGGTCTGTCCCCTCCATTGGCATGTCTGTCATCGTCTCCCACTGAA

CCCCAAAGTTCTGATAGGATTTGGGGCACCATATGAGGAATTCAGTTGCCTTAGCTTGAA

ACTTATCCGATCTGATCTGCAAATAGGACACGGCCTTTGAAGGTCCTGCTGGGTGACCCC

AATCCTTGTGCTGTTGACCACAGAGAAAAGAGTGGGGCCGAGCACTGCATTTATGAGAAT

TTGTCTCCACTTTGTCAGAGATCTCTGTGTGAAGTACACTGTTCTCATCCCCACCAAGCT

CCCAGCTCAGCAGTGTTTTCTGCACTTGGGCTGGTTGGATGGAGGACAAGTCAGAGAACT

**Data S1. Consensus sequence of alternatively spliced ZDHHC6 produced using a genome guided de-novo assembly with Trinity**
